# Nonhuman Primate Models for SARS

**DOI:** 10.1371/journal.pmed.0030194

**Published:** 2006-04-18

**Authors:** Bart L Haagmans, Albert D. M. E Osterhaus

## Abstract

Osterhaus and Haagmans discuss a new study in *PLoS Medicine* that supports the use of the cynomolgus macaque model to study SARS pathogenesis and to test intervention strategies.

Severe acute respiratory syndrome (SARS) first emerged in Guangdong Province, the People's Republic of China, in November 2002. The disease was characterised by a rapidly progressive atypical pneumonia [ 1]. In addition to other pathogens, a coronavirus not previously discovered was identified in patients with SARS. Reproduction of a similar disease in cynomolgus macaques with this virus completed Koch's postulates, implicating SARS coronavirus (SARS-CoV) as the aetiologic agent [ 2, 3].

## Pathogenesis of SARS

In typical cases, which were largely confined to adult and elderly individuals, SARS presented with acute respiratory distress syndrome (ARDS)—characterised by the presence of diffuse alveolar damage (DAD) upon autopsy. The pathological changes in lung alveoli most likely follow a common pathway characterised by an acute phase of protein-rich alveolar fluid influx into the alveolar lumina as a consequence of the injury to the alveolar wall. Subsequently, type-2 pneumocyte (see Glossary) hyperplasia occurs to replace the loss of infected type-1 pneumocytes and to cover the denuded epithelial basement membrane, resulting in restoration of the normal alveolar architecture. Severe alveolar injury may lead to fibrosis with loss of alveolar function in more protracted cases.

SARS-CoV was detected not only in the respiratory tract but also in faeces and urine of patients. Replication of the virus in the gastrointestinal tract was confirmed by electron microscopic studies of biopsies of the upper and lower intestinal mucosae of patients with SARS. Faecal transmission proved to be important in at least one major community outbreak in Hong Kong, in which over 300 patients were infected within a few days [ 1].

## Animal Models for SARS

Development of animal models for SARS-CoV infection of humans is of utmost importance to elucidate the pathogenesis of SARS and to develop intervention strategies against the infection. A wide range of animal species is susceptible to experimental infection with SARS-CoV, including rodents (mice and hamsters), carnivores (ferrets and cats), and nonhuman primates (cynomolgus and rhesus macaques, common marmosets, and African green monkeys) [ 3–11]. Adult mice infected with varying doses of SARS-CoV in the respiratory tract show no clinical signs of disease, although the virus replicates in respiratory tissues, peaking early after infection, with viral titres in the lungs reaching relatively high levels. The infection is accompanied by only mild inflammatory changes of the respiratory tract. On the other hand, aged mice, as well as hamsters and ferrets, do show signs of clinical disease (weight loss and ruffled fur), albeit, in most cases, in the absence of the typical lung lesions seen in humans with SARS [ 4–6].

In contrast, SARS-CoV inoculation in the respiratory tract of cynomolgus macaques causes infection of bronchial epithelial cells, type-1 and type-2 pneumocytes at one to four days postinfection, followed by extensive type-2 pneumocyte hyperplasia in the lungs at four to six days postinfection [ 3, 11]. Moreover, multiple foci of acute DAD are observed, characterised by flooding of alveoli with protein-rich oedema fluid mixed with variable numbers of neutrophils and rare syncytia ( Figure 1), and by extensive loss of epithelium from alveolar and bronchiolar walls. These lesions are quite similar to those observed in humans in the acute stages of SARS. Although clinical signs (respiratory distress and general malaise) were observed, they were not further studied in the initial experiments.

**Figure 1:**
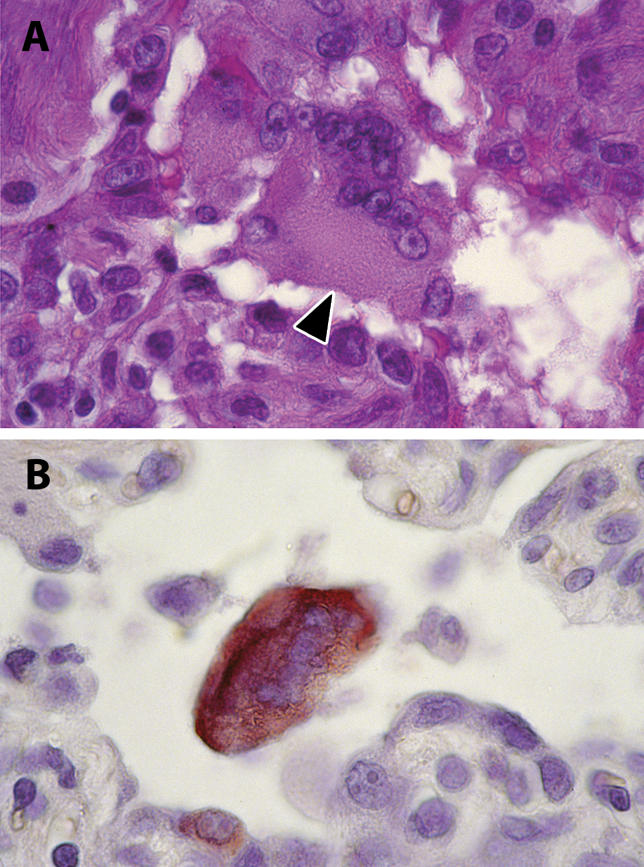
Histologic Lesions in Lungs from Cynomolgus Macaques Experimentally Infected with SARS-CoV Syncytia (indicated by the arrowhead) in the lumen of a bronchiole (A) and expression of SARS-CoV antigen by a syncytium in the lumen of an alveolar duct (B). (Figure derived from Figures 1 and 3 in [ 3]; Photos: Thijs Kuiken)

Studies from different laboratories confirmed that nonhuman primates could be infected experimentally with SARS-CoV, although the severity of the induced lung pathology varied and clinical signs were generally not reported. So far, factors underlying these differences have not been delineated, but virus strain, age, microbiological state, genetic background, and origin of the animals could have played a role.

In this issue of *PLoS Medicine,* James Lawler, Jason Paragas, and colleagues provide further support for the use of the cynomolgus macaque model to study the pathogenesis of SARS and to test intervention strategies [ 12]. They show that infection of these macaques with a molecularly cloned SARS-CoV resulted in a mild-to-moderate symptomatic illness, indistinguishable from the illness induced with wild-type SARS-CoV. Chest radiographs from several infected animals showed unifocal or multifocal pneumonia that peaked between eight to ten days postinfection. The authors point out that the results are reminiscent of the milder syndrome observed in younger humans infected with SARS-CoV. Molecular modification of the cloned virus may now further allow deciphering of the role of different viral gene products in the pathogenesis of SARS. An important result of this present study is the observation that one group of animals inoculated via the nasal and ocular route exhibited significant faecal shedding of SARS-CoV. Thus far, efforts to evaluate the efficacy of SARS-CoV vaccines have largely aimed at protecting the respiratory tract.

## Implications for Future SARS Research

The persistent threat of a possible new introduction of SARS-CoV or of a related virus in the human population necessitates further refinement of animal models to elucidate the pathogenesis of, and intervention strategies against, SARS. Although none of the current animal models have fully reproduced all features of SARS, the most important aspects of SARS are observed in experimentally infected nonhuman primates. Such models have already demonstrated the protective effect of pre- and postexposure use of pegylated human interferon-α, of candidate SARS-CoV vaccines, and of convalescent sera from SARS patients (unpublished results; [ 10, 11]). In the absence of ongoing human SARS-CoV infections, candidate SARS vaccines may have to be approved on the basis of animal data alone, preferably in two different species (the “two animal rule”). But in order for such approval, the pathophysiological mechanisms underlying SARS pathogenesis and the preventive efficacy of such vaccines need to be well understood. The present study further supports the use of nonhuman primate SARS models for pathogenesis studies and for the development of intervention strategies. Infection of cynomolgus macaques with recombinant SARS-CoV containing defined modifications may allow further elucidation of SARS.

**Glossary**

**Type-1 Pneumocytes:** These cells are responsible for alveolar gas exchange. They cannot replicate and are susceptible to a large number of toxic insults.

**Type-2 Pneumocytes:** These cells are responsible for producing and secreting surfactant. They can replicate in the alveoli to replace damaged type-1 pneumocytes.

## Abbreviations

ARDS: acute respiratory distress syndrome
DAD: diffuse alveolar damage
SARS: severe acute respiratory syndrome
SARS-CoV: SARS coronavirus
